# The B Lymphocyte Differentiation Factor (BAFF) Is Expressed in the Airways of Children with CF and in Lungs of Mice Infected with *Pseudomonas aeruginosa*

**DOI:** 10.1371/journal.pone.0095892

**Published:** 2014-05-21

**Authors:** Daniel R. Neill, Gemma L. Saint, Laura Bricio-Moreno, Joanne L. Fothergill, Kevin W. Southern, Craig Winstanley, Stephen E. Christmas, Joseph R. Slupsky, Paul S. McNamara, Aras Kadioglu, Brian F. Flanagan

**Affiliations:** 1 Department of Clinical Infection, Microbiology and Immunology, Institute of Infection and Global Health, University of Liverpool, Liverpool, United Kingdom; 2 Department of Women's and Children's Health, Institute of Translational Medicine, University of Liverpool, Alder Hey Children's NHS Foundation Trust Hospital, Liverpool, United Kingdom; 3 Molecular and Clinical Cancer Medicine, Institute of Translational Medicine, University of Liverpool, Liverpool, United Kingdom; University of Pittsburgh, United States of America

## Abstract

**Background:**

Chronic lung infection with *Pseudomonas aeruginosa* remains a major cause of mortality and morbidity among individuals with CF. Expression of mediators promoting recruitment and differentiation of B cells, or supporting antibody production is poorly understood yet could be key to controlling infection.

**Methods:**

BAFF was measured in BAL from children with CF, both with and without P. aeruginosa, and controls. Mice were intra-nasally infected with *P. aeruginosa* strain LESB65 for up to 7 days. Cellular infiltration and expression of B cell chemoattractants and B cell differentiation factor, BAFF were measured in lung tissue.

**Results:**

BAFF expression was elevated in both *P. aeruginosa* negative and positive CF patients and in P. aeruginosa infected mice post infection. Expression of the B cell chemoattractants CXCL13, CCL19 and CCL21 increased progressively post infection.

**Conclusions:**

In a mouse model, infection with P. aeruginosa was associated with elevated expression of BAFF and other B cell chemoattractants suggesting a role for airway B cell recruitment and differentiation in the local adaptive immune response to *P. aeruginosa*. The paediatric CF airway, irrespective of pseudomonal infection, was found to be associated with an elevated level of BAFF implying that BAFF expression is not specific to pseudomonas infection and may be a feature of the CF airway. Despite the observed presence of a potent B cell activator, chronic colonisation is common suggesting that this response is ineffective.

## Introduction

Chronic lung disease is the major cause of morbidity and mortality among individuals with Cystic Fibrosis [CF]. Prominent among the causes of such lung disease is chronic infection with the opportunistic pathogen *Pseudomonas aeruginosa* [1]. Immune defence against respiratory tract pathogens is dependent upon a complex interplay of both innate and adaptive components that may prevent or clear infection. However CF is a leading example of an illness in which this complex interplay between innate and adaptive immunity is not protective as evidenced by long term P. aeruginosa infection.

Production of pathogen-specific antibody by B lymphocytes is normally an important mechanism of airway defence against *P*. aeruginosa infection [2], [3]. Studies, in mice and humans, have characterised the specificity and magnitude of antibody responses to *P*. aeruginosa infection [3] and notably antibody production to vaccine candidates has been correlated with protection upon subsequent *P. aeruginosa* challenge [4] but antibody production does not correlate with protection in the CF airway.

To improve strategies for airway vaccine design and new therapeutics against *P. aeruginosa*, a better understanding of antibody induction in the airways is needed. However, the mediators that promote recruitment and differentiation of B cells to the lung or support airway antibody production during infection are poorly understood, both with respect to *P. aeruginosa* and airway pathogens in general [5]. We have previously reported production of the B cell differentiation factor, B cell activating factor of the TNF family [BAFF, also known as BLys or TNFS13B] by human lung epithelia in response to viral infection [6]. Studies using influenza infected mice show that homeostatic chemokines including CXCL13, CCL19 and CCL21 are essential for effective pulmonary defence, regulating B and T cell recruitment to the airway, stimulating dendritic cells and influencing formation of inducible bronchial associated lymphoid tissues [iBALT] [7]. In mice infected with *P. aeruginosa*, Eppert et al showed a possible role for CCL19 and CCL21 in regulating lymphocyte or dendritic cell recruitment but only examined one day post infection [8]. The BAFF response to ongoing airway *P. aeruginosa* infection has not been studied, but recombinant BAFF, used as an adjuvant, has been shown to enhance the airway response to heat-killed *P. aeruginosa* in mice [9].

We firstly sought to establish if expression of BAFF is a feature of the airway response to chronic bacterial infection, including *P. aeruginosa*, analysing lower airway samples from children with CF, with and without *P. aeruginosa* infection, compared to healthy control patients. To validate our findings further we used a 7-day murine respiratory inhalation model to establish stable infection of the airways and study the inflammatory response to *P. aeruginosa*, specifically with regards to the recruitment and potential stimulation of B cells. In our murine respiratory inhalation model, mice are challenged with the clinically relevant *P. aeruginosa* Liverpool epidemic strain [LES] isolate LESB65, which establishes a stable 7 day infection that is restricted to the airway [10]. In contrast to models using non-colonising *P. aeruginosa* strains that are rapidly cleared by components of the innate immune system or more aggressive strains that cause sepsis and host death, this model allows the lung response to *in situ* infection to develop over a longer time period, such as occurs in CF [10].

Our results demonstrate for the first time the kinetics of B cell chemoattractant and differentiation factor responses both following *P. aeruginosa* infection and in relation to lung B cell recruitment in a murine model. An elevated level of BAFF was found to be associated with the paediatric CF airway, irrespective of the presence or absence of pseudomonal infection, implying that the expression is not specific to pseudomonas infection and may be a feature of the CF airway. The importance of the B cell response in the CF lung remains unclear and its effectiveness is complicated by other factors predisposing to poor bacteria eradication such as impaired mucocilary clearance.

## Patients and Methods

### Patient samples

Bronchoalveolar lavage [BAL] was obtained bronchoscopically from CF patients who were being investigated electively for persistent respiratory symptoms. Lower respiratory lavage fluid was obtained non-bronchoscopically from the remainder of CF patients and non-CF, healthy control patients, when anaesthetised for routine elective surgery as described previously [11]. Microbiological analysis was conducted on BAL from all CF patients in the microbiology department of this specialist hospital [BAL from non-CF healthy control children was not sent for analysis]. Lavage fluid was aliquoted and stored at −70°C until used for analysis.

### Ethics Statement

The paediatric ethics committee in Liverpool approved the study and all samples were collected following written informed consent from parents [REC:]06/Q1502/142].

### Mice

Female BALB/cOlaHsd mice were purchased from Harlan Laboratories [Bicester, UK] and acclimatised for one week prior to use. Mice used for infection experiments were between 9 and 12 weeks of age and were age matched for each experiment. The U.K. Home Office approved the experimental protocols.

### Infection of Mice

Animals were anaesthetised with a mixture of oxygen and isofluorane and infected intranasally with 1×10^6^ CFU *P. aeruginosa* LESB65 in 50 µl phosphate buffered saline [10].Mice did not develop visible signs of disease and were culled at pre-determined times post-infection by cervical dislocation. Lungs were removed and either homogenised for CFU counts and ELISA or prepared for flow cytometry by incubation for 30 min at 37°C with 10 mg/ml collagenase D [Amersham], followed by homogenization and lysis of erythrocytes [12].

### Bacterial Counts

Serial dilutions to 10^−7^ made using 20 µl samples of homogenised lung were analysed in sterile 96-well microtiter plates [GIBCO, Paisley, United Kingdom] containing 180 µl of sterile nanopure water per well. Pseudomonas selective agar plates were marked into six sectors, and three 20 µl aliquots of each dilution were plated into each sector; this procedure was repeated in duplicate. Once dry, plates were incubated at 37°C overnight. Colonies were counted in sectors containing a measurable number [approximately <300].

### Flow cytometry

Mouse tissue cell suspensions, at 2×10^8^ cells/ml, were incubated with purified anti-Fc receptor blocking antibody [anti-CD16/CD32] before addition of the specific antibodies. Cell surface markers were stained using a combination of fluorescein isothiocyanate [FITC]-, phycoerythrin [PE]-, PE-Cy7- and allophycocyanin [APC]-conjugated monoclonal antibodies directed against: T-cells CD3 [clone 17A2], CD4 [GK1.5], CD8 [53-6.7], B-cells CD19 [1D3], Neutrophils Gr-1 [RB6-8C5], Macrophages F4/80 [BM8]. Where appropriate, CD45 [30-F11] was used to identify haematopoietic cells. In each experiment the appropriate isotype control monoclonal antibodies and single conjugate controls were also included. All antibodies were purchased from eBioscience. Samples were analysed using a Becton Dickinson FACScalibur flow cytometer running CellQuest acquisition and analysed using FlowJo software [version 8.8.3, Tree Star].

### ELISA assays

Lung homogenates were prepared in ice cold PBS containing a protease inhibitor cocktail before being clarified by centrifugation for five minutes at 4°C. Total protein concentrations were determined by using a DC protein assay kit [Biorad] according to the manufacturer's instructions and expression per milligram of lung homogenate calculated. Chemokines CXCL13, CCL19 and CCL21 were measured using DuoSet ELISA assays as described by the manufacturer [R&D systems] and both murine and human BAFF using quantikine ELISA kits [R&D systems].

### Statistical Analysis

Data were analyzed using independent samples *t* tests or Mann-Whitney *U* tests depending on data distribution determined by the 1-sample Kolmogorov-Smirnov test. Data were analyzed using SPSS 20 software [SPSS Inc].

## Results

### BAFF Expression in Cystic Fibrosis Patient Bronchoalveolar Lavage

**Figure 1:**
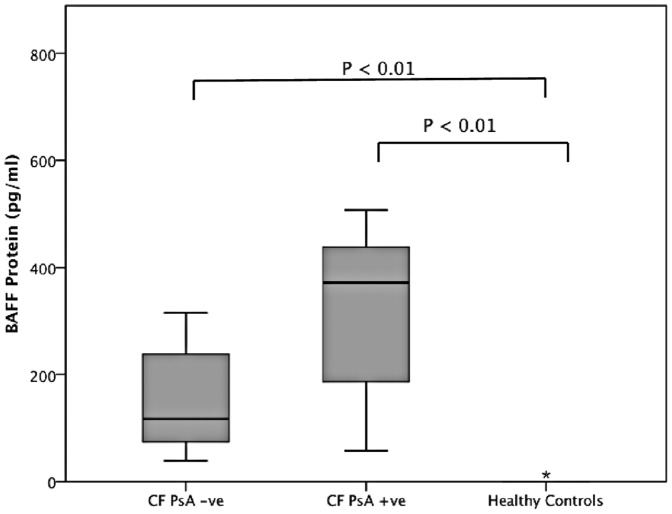
BAFF expression in BAL fluid. Expression was measured by ELISA in BAL fluid from *P. aeruginosa* positive [n = 6] and negative [n = 14] CF patients and healthy controls [n = 7]. BAFF expression was significantly elevated [p<0.01] in both the PA infected group and non PA infected group in comparison to the control group.

To determine if BAFF expression is a characteristic of human lung bacterial infection, BAFF was measured in BAL from 20 school age children with CF [mean [SD] age, 8.0 [4.5] years]. Samples from six of these children were obtained bronchoscopically when they were being investigated for persistent respiratory symptoms. Samples from the remaining 14 CF patients and 7 healthy control children [6.8 [3.5] years] were obtained non-bronchoscopically when these children attended hospital for routine elective surgery. BAL from six of the CF patients cultured *P. aeruginosa*, with the remainder being culture positive for other organisms including *Haemophilus influenzae*, *Stenotrophomonas maltophilia* and *Aspergillus* species [n = 6] or BAL fluid culture negative [n = 8] [a full list of all organisms is available in Table S1]. BAFF expression was significantly elevated in both *P. aeruginosa* positive and negative patient groups [p<0.01] compared to the control group [Figure 1].

### Bacterial Infection and Cellular Infiltration in a murine respiratory inhalation model of *P. aeruginosa* infection

**Figure 2:**
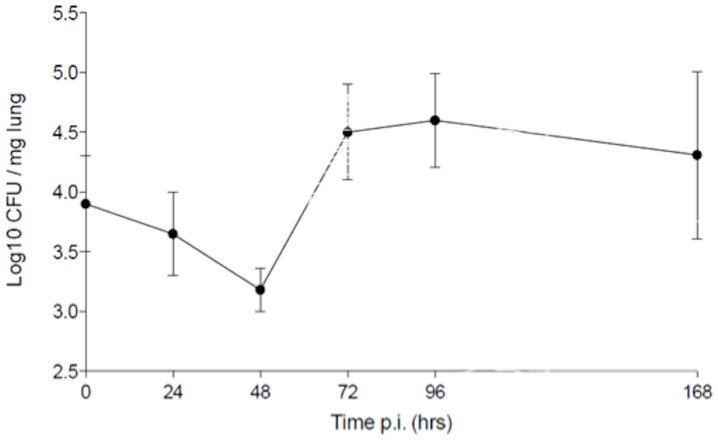
Kinetics of airway infection by *P. aeruginosa* LESB65. Mice were challenged [intranasal] with 10^6^ CFU. Values shown as an average Log CFU/mg of lung tissue homogenised, n = 5 mice at each time point. Time zero samples were taken at 15 minutes post infection to confirm entry into the lung and determine levels immediately post infection.

A murine respiratory infection model was used to assess the inflammatory response to stable *P. aeruginosa* LESB65 strain infection in lungs. Balb/c mice were challenged intranasally and lung bacterial load and inflammatory cell infiltration monitored over a 7 day period post-infection. As shown in Figure 2, challenge with LESB65 resulted in stable infection of the bacteria within the lung with CFU/mg of tissue initially Log 4, Log 3.2 at 48 hours and Log 4.5 at day 7 post-challenge. LESB65 was restricted to the lungs and no bacteria were recovered from blood at any time point post infection.

**Figure 3:**
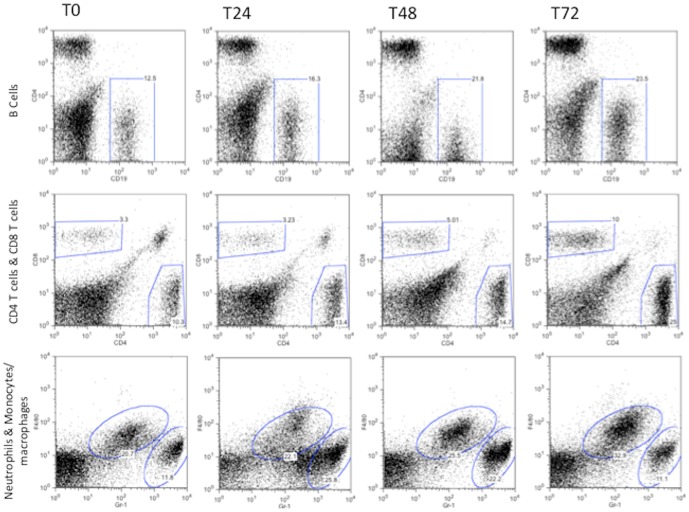
FACS analysis of lung inflammatory cells following *P. aeruginosa* LESB65 infection of mice. Representative FACS plots showing immune cell staining of airway cells. Each column shows plots from an individual mouse at each time point. All plots are lung CD45+ cells and gates were drawn relative to isotype control antibody staining for each marker. Top row shows B cells [CD45+, CD19+], middle row shows CD4 T cells [CD45+, CD3+, CD4+] and CD8 T cells [CD45+, CD3+, CD8+], bottom row shows neutrophils [CD4+, Gr-1hi, F4/80-] and monocytes/macrophages [CD45+, F4/80+, Gr-1int/low]. Numbers on gates are percentages of total CD45+ cells.

**Figure 4:**
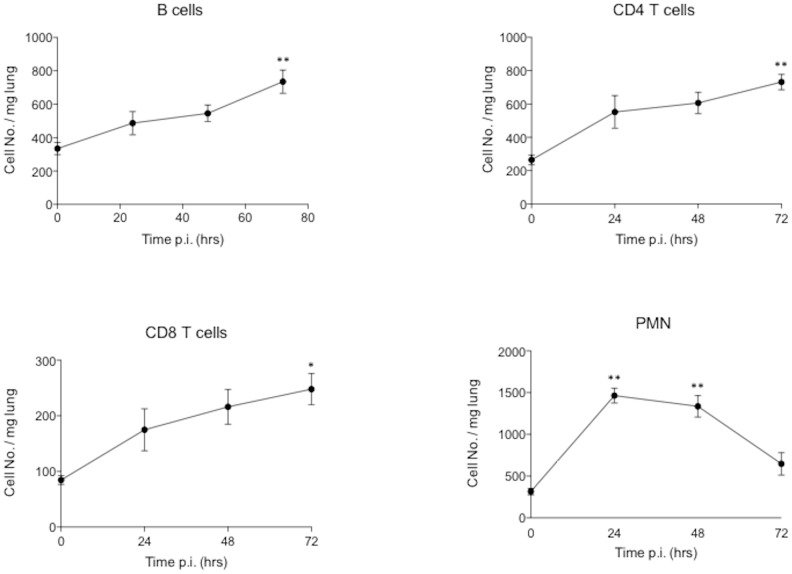
Airway leukocyte number following LESB65 infection. Average B lymphocyte, CD4+ve T cell, CD8+ve T cell and neutrophil count shown as mean and standard deviation per mg of tissue at 24, 48, and 72 hours post infection [N = 5 each time point]. * = p<0.05, ** = p<0.01.

Measurement of the inflammatory cell response showed the predominant cell infiltrating the lung at early time points to be neutrophils. A peak of 1500 cells/mg tissue being obtained at 24 hours post infection with significantly elevated numbers present at 24 hours [P<0.01] and 48 hours [P<0.01] but not at 72 hrs [Figure 3 and 4]. In contrast, B cells, and CD4 and CD8 T lymphocyte numbers increased with significantly elevated numbers at 72 hours post infection [B cells P<0.01, CD4 P<0.01, CD8 P<0.05] with CD4 cells being the predominant T cell type [Figure 3 and 4]. B lymphocytes and CD4 T cells numbers reached 800 cells/mg of lung tissue by 3 days post-infection [Figure 3 and 4] with CD8 cell numbers being consistently lower.

### Expression of mBAFF and Lymphoid Cell Chemoattractants CXCL13, CCL19 and CCL21

**Figure 5:**
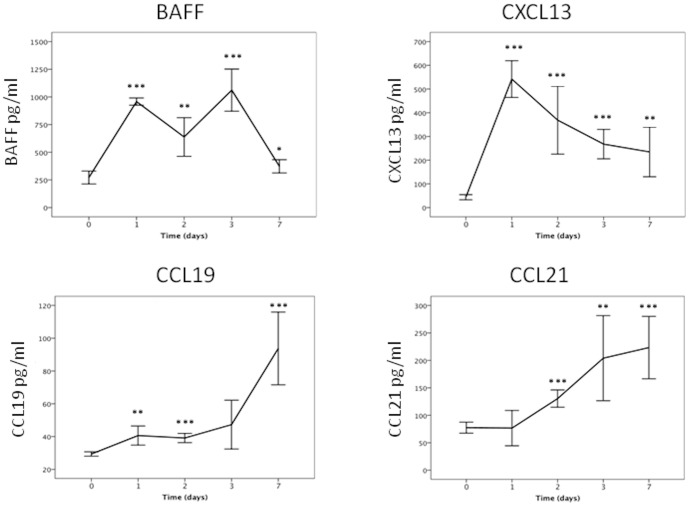
Expression of BAFF, CXCL13, CCL19 and CCL21 in mouse lung tissue. Each cytokine was measured at days 1, 2, 3 and 7 days after infection with 10^6^ CFU of *P. aeruginosa* LESB65 by ELISA. [n = 5]. BAFF was significantly expressed at days 1 [p<0.001], 2 [p<0.01], 3 [p<0.001], and 7 [p<0.05] in comparison to day 0 control. CXCL13 at days 1, 2, 3, [all p<0.001] and 7 [p<0.01]. CCL19 at days 1 [p<0.01], 2 [p<0.001], and 7 [p<0.001]. CCL21 at days 2 [p<0.001], 3 [p<0.01] and 7 [p<0.001]. * = p<0.05, ** = p<0.01, *** = p<0.001.

To determine if increased expression of BAFF is also a feature of the response to airway *P. aeruginosa* infection in this murine model system, mean [SD] protein levels were measured in lung homogenate samples prepared from mice at day 0 and at 1, 2, 3 and 7 days post infection [all n = 5]. BAFF was found to be significantly elevated at day 1 [892 [199] pg/ml p<0.001], day 2 [750 [242] pg/ml p<0.01], day 3 [1081 [214] pg/ml p<0.001] and day 7 [386 [64] pg/ml p<0.05] [Figure 5].

Expression of the chemoattractants CXCL13, CCL19 and CCL21 were measured to assess how B lymphocytes could be recruited into the lung [Figure 5]. Highest expression of CXCL13 was observed on days 1 [Mean [SD], 521 [179] pg/ml] and 2 [523 [152] pg/ml] post infection with levels decreasing progressively to day 7 when they were still above control values [P<0.005]. In contrast, CCL19 and CCL21 expression reached peak values, mean [SD], at day 7, [94 [24] pg/ml and 223 [64] pg/ml respectively], CCL19 being significantly elevated compared to control from day 1, mean [SD] 204 [6.5] pg/ml, [p value <0.01], but CCL21 being significantly elevated only from day 2 onwards [P<0.001].

## Discussion

Although many studies have focused on the mechanism and importance of inflammatory cell recruitment into the lung following *P. aeruginosa* infection, few studies have examined the B cell response within the airway. Here we demonstrate, to our knowledge for the first time, that BAFF expression is increased in BAL from the lungs of patients with CF. Furthermore, using a respiratory inhalation model of stable long-term lung infection with *P. aeruginosa* LESB65 in mice, we show, to our knowledge for the first time, recruitment of both B and T lymphocytes, expression of the B cell chemo-attractant cytokines CXCL13, CCL19 and CCL21 and up-regulation of the B cell differentiation factor BAFF.

We have previously shown that the B cell differentiation factor BAFF, a major B cell growth factor that promotes B cell maturation and antibody production is expressed in the human airway during a range of viral infections including respiratory syncytial virus [6]. To determine if BAFF expression is a component of the human airway response to *P. aeruginosa* infection, we measured BAFF expression in BAL from children with CF both with and without *P. aeruginosa* infection. Both groups showed significant elevation in BAFF expression in comparison to the control group. The *P. aeruginosa* negative CF patient group, some of whom were culture positive for other bacterial infections, also showed elevated BAFF expression, indicating that this response is not *P. aeruginosa*-specific. The recent observation that BAFF is expressed during Mycobacterial infection is consistent with this suggestion [13]. Furthermore, our previous finding that BAFF expression occurs in response to a range of viral infections [6], supports this being a general mechanism in airway defence. The findings presented here cannot discount the possibility that elevated BAFF expression may be an innate characteristic of the CF airway associated with the underlying CFTR defect or of immune or inflammatory processes within the CF airway.

To further determine if expression of this B cell differentiation factor correlated with increased expression of B cell recruitment factors and increased B cell numbers in the lung, we used an animal model of chronic pseudomonas infection. Neutrophils were the major inflammatory cell recruited to the lung, with numbers peaking at day 1 post-challenge before reducing at days 2 and 3. The 24–48 hour peak of neutrophil influx coincides with a significant drop in bacterial load in the lung and the subsequent decline in lung neutrophil numbers is matched by a corresponding steady decrease in bacterial CFUs. This pattern of rapid neutrophil influx and gradual decline is in keeping with other respiratory pathogen infection models of the lungs e.g. streptococcus pneumonia, where early and rapid neutrophil influx is ineffectual in clearance and containment of bacterial loads [14]. In contrast, B cell and T cell [both CD4 and CD8] influx into the lung progressively increased over the study period of 7 days post-infection. To our knowledge no previous studies have documented B cell recruitment into the lung following *P aeruginosa* infection. In contrast, T cell responses have been studied with CD4 cell Th2 and Th17 type responses being described in human *P. aeruginosa* infection [15]–[17], while CD4 Th1 cells have been reported to be protective in mice [18].

In order to understand better how the observed recruitment of B and T cells into the lungs is mediated, we looked at expression of the homeostatic chemokines, CXCL13, CCL19 and CCL21, known to influence lymphocyte migration and to be expressed by epithelial, endothelial and dendritic cells [7], [19]. Increased CXCL13 expression from day 1 post infection preceded increased CCL19 and CCL21. Elevation of CXCL13, primarily a B cell chemoattractant with highest expression on days 1 and 2 coincided with the increase in B cell numbers, suggesting that this chemoattractant may be important in the local B cell response to *P. aeruginosa*, possibly supporting the recruitment of both B1 and B2 B cell types [19].

In contrast, expression of the B cell, T cell and DC chemoattractants CCL19 and CCL21 increased progressively to 7 days post infection with CCL19 elevated at day 1 and rising before CCL21. A previous study, using the PAO1 strain, reported increased expression of CCL19 but not CCL21 on day 1 following *P. aeruginosa* infection but did not study later time points [8]. The data presented here show that both are upregulated post infection but with differential kinetics, CCL19 levels initially being increased before CCL21, although CCL21 is expressed at higher levels by day 7. Our data supports a role for these two chemokines in later recruitment of B and T lymphocytes and potentially DCs whilst CCL19 could also have a role in the early recruitment of B cells. CXCL13, CCL19 and CCL21 have previously been shown to be essential components of the local airway immune response to viral infection [7] and to be involved in iBALT formation, a recognised feature of long term pathogen exposure [20]. It is possible that recruitment of both B and T cells seen in this study represents the early stages of iBALT formation and the induction of a local adaptive immune response.

Our results demonstrate that increased BAFF expression is a characteristic of the airway immune response at similar time points to increased expression of B cell chemoattractant and elevated B cell numbers. This suggests that not only are B cells recruited to the airway but that the local environment is capable of supporting B cell survival, differentiation and antibody production.

That airway BAFF can act to promote antibody secretion is supported by previous work showing BAFF, when used as an adjuvant in mucosal immunisation with heat killed Pseudomonas, resulted in increased antibody production [9]. The mechanism leading to expression of BAFF in the mouse airway in response to *P. aeruginosa* has not been defined here. In viral infection it is expressed by infected epithelial cells and dependent on interferon beta production [6], [21]. Airway epithelial cells from CF patients have been shown to produce less interferon in response to viral infection [22], [23]. If a similar mechanism occurs in response to bacterial challenge, reduced levels of BAFF and consequent lower local antibody production may be a further factor compromising airway defence in this patient group.

Collectively our results demonstrate differential expression of both B and T lymphocyte chemoattractants, recruitment of B cells and significant expression in both mouse and humans of the key differentiation factor BAFF, potentially capable of supporting B cell maturation in the lung.

## Supporting Information

Table S1 **Additional Bacteria identified in patient BAL samples and respective P.** ***aeruginosa*** **status for each patient.**
(TIFF)
