# Supplementary figures and images for: The B Lymphocyte Differentiation Factor (BAFF) Is Expressed in the Airways of Children with CF and in Lungs of Mice Infected with Pseudomonas aeruginosa
Source: PLoS One. 2014 May 21;9(5):e95892. doi: 10.1371/journal.pone.0095892 (PMC4029587; doi:10.1371/journal.pone.0095892)

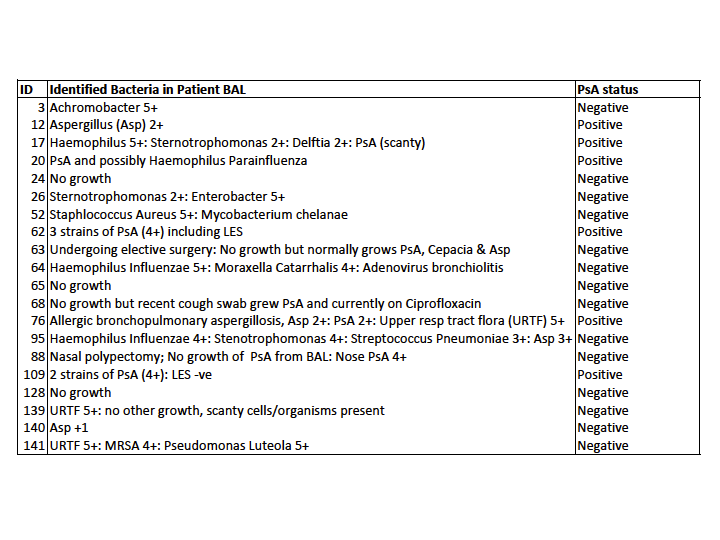

Supplement: Table S1 — Additional Bacteria identified in patient BAL samples and respective P. aeruginosa status for each patient. (TIFF) [file pone.0095892.s001.tiff]
